# Home to Hospital Live Streaming With Virtual Reality Goggles: A Qualitative Study Exploring the Experiences of Hospitalized Children

**DOI:** 10.2196/pediatrics.9576

**Published:** 2018-12-13

**Authors:** Aafke Bakker, Lindy Janssen, Cees Noordam

**Affiliations:** 1 Amalia Children's Hospital Department of Pediatrics Radboud University Medical Center Nijmegen Netherlands

**Keywords:** experiences, hospitalization, mobile phone, livestream, pediatrics, qualitative analysis, videoconferencing, virtual reality

## Abstract

**Background:**

Being separated from home and relatives is a major stressor for children and adolescents when hospitalized. Children long for a manner to be distracted, pleasured, and socially connected during hospitalization. Different technological devices have been applied in health care to answer those needs. Both virtual reality (VR) and videoconferencing have proven their value in hospital wards and pediatrics. VisitU combines these 2 technologies innovatively. VisitU is a recently launched VR product enabling users to be virtually at home during hospitalization.

**Objective:**

This study aims to explore the experiences of hospitalized patients with the VR intervention of VisitU in addition to standard care.

**Methods:**

Over a 3-month period, a purposive sample of 10 patients hospitalized in the Radboudumc Amalia Children’s Hospital was included in this qualitative study. Semistructured interviews were performed, one before and one after the use of the VR device. Patients were asked open-ended questions concerning their experiences with VisitU on practical, cognitive, emotional, and social domains. The interviews were audiorecorded and transcribed verbatim. Atlas.ti was used to support the qualitative analysis. Furthermore, the inductive thematic analysis was done according to the 6-step procedure described by Braun and Clarke.

**Results:**

The following 6 main themes were the result of the qualitative analysis: “Being hospitalized,” “Expectations of VisitU,” “VisitU in use,” “VisitU, the benefits,” “The impact of VisitU,” and “Barriers when using VisitU.” The way VisitU was used by patients varied. The main benefits of VisitU were being somewhere else, being at home, and facilitating social connection. Limitations were experienced on the technical abilities, physical side effects, and complexity of use. Despite that, patients were positive about VisitU and unanimous in the view that they would like to use it again and advise other patients to use it.

**Conclusions:**

This study shows the positive experiences of pediatric patients with VR live streaming. VisitU brings together the needs of patients and possibilities of VR and videoconferencing; it offers patients a way out of the hospital. Nevertheless, practical and technical obstacles must be overcome and side effects are an area of further research.

## Introduction

Hospitalized children and adolescents have to cope with a complete change in their environment, people around them, and daily activities [1]. Factors found to influence their experience with hospitalization have been explored in several studies. Loneliness and boredom are 2 themes frequently mentioned [2-6]. In every age group, children reported feeling separated from home, family, and friends as one of the worst experiences during hospitalization [1,3,5,6]. Therefore, they long to communicate with peers and maintain contact with the world outside the hospital [2,4]. In addition, some patients miss appropriate toys and amusement. To oppose boredom, younger children show the desire to play and be entertained by videos or games, whereas teenagers prefer entertainment designed for their own age groups [3,5]. In search of answering those calls for connection, distraction, and pleasure in the hospital, different technological devices have been applied in health care during the last decade.

Both videoconferencing and virtual reality (VR) have been utilized to improve hospitalization and health care. Videoconferencing is defined as a live meeting of 2 or more people in separate locations being connected audiovisually through a computer or smartphone. Quantitative and qualitative studies have investigated the use of videoconferencing for hospitalized patients to keep in touch with their family, friends, or classmates [7-9]. In a qualitative study, the ability to communicate was highlighted as a primary benefit, and parents described a marked improvement of patients’ mood [7]. Nicholas et al reported that the application of videophones decreased feelings of isolation and anxiety and increased feelings of connection between family members [8]. Yang et al evaluated the effect of videoconferencing during hospitalization on the reduction of stress experienced by children; their study demonstrated that the use of videoconferencing is associated with greater reduction of stress compared with those who do not use videoconferencing [9].

In addition, VR is a promising technology in health care. It is defined by the British dictionary as a computer-generated environment that closely resembles reality to the person experiencing it [10]. The virtual environment nowadays is mainly obtainable through a smartphone placed in a head-mounted display. The view to the real environment is cut off by the goggles, and patients are only able to look into the virtual world [11,12]. A considerable amount of literature has been published on the use of VR in reducing pain [13-21]. Malloy and Milling showed in a systematic review that VR distraction is an effective intervention for experimental pain and pain associated with burn injuries [14]. Especially with pediatric patients, Hua et al found a marked reduction in pain scores and heart rate when VR distraction was used in the treatment of chronic wounds [16]. Together, these studies indicated that VR is an effective distracter and a promising nonpharmacological analgesic intervention. Most studies illustrated the use of VR in outpatient settings instead of hospitalized patients [12,22,23]. However, VR in a hospital ward seems feasible and without great side effects [22-25]. A recent systematic review of VR for medical inpatients found it to be efficacious, easy to use, safe, and contributing to patient satisfaction [22]. Among oncological inpatients, studies investigating VR reported improvement of emotional state and positive emotions [12].

In the last few years, the costs of VR technology have decreased, and VR devices have become widely available and affordable. In addition to other VR devices, VisitU launched a VR technology enabling users to be virtually at home during hospitalization. The livestream connection provides patients with a 360° look around their home and a live chat with their relatives [26]. VisitU is a recent product that innovatively combines videoconferencing and VR to satisfy the need to connect with home and relatives.

As mentioned above, children report separation from family and friends as a major stressor during hospitalization. Therefore, VisitU is worth being investigated. This would be the first study that focuses on VR live streaming, the combination of videoconferencing and VR in one device. The objective of this study is to investigate the experience with VisitU among hospitalized children and adolescents. To explore the first experiences with VisitU, a qualitative approach was chosen.

## Methods

### Setting and Sample

Over a 3-month period (June-August 2017), eligible patients in the Radboudumc Amalia Children’s Hospital (Nijmegen, the Netherlands) were included in this qualitative study. Children were aged 6-18 years and hospitalized on the medium care unit for at least 4 days. Children with an increased risk of seizures, severe visual impairment or blindness, reduced consciousness, severe mental retardation, or non-Dutch-speaking were excluded [27]. A purposive sample of patients was recruited to achieve a range in age, gender, hospital to home distance, and “hospital experience.” One after another, patients were selected from the eligible children. Data saturation and research period determined the sample size. Data saturation was reached when no new topics were discussed during the interviews. This study was approved by the Research Ethics Committee of the Radboud University Nijmegen Medical Centre. The study did not fall within the remit of the Medical Research Involving Human Subjects Act (WMO).

**Figure 1:**
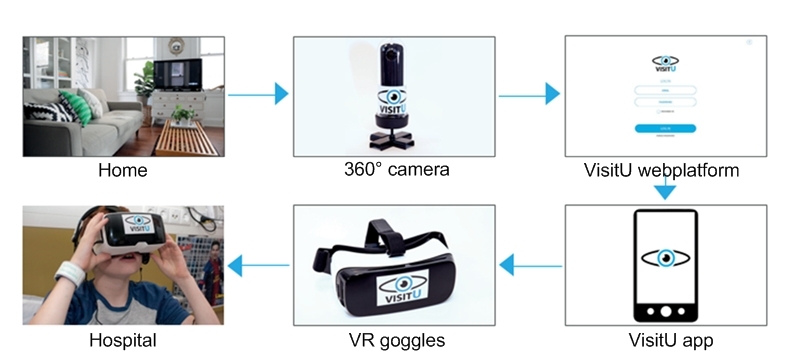
VisitU home to hospital livestream.

### Intervention

VisitU includes an Asus Zen Book UX305 with video card and video live streaming software, a 360° Theta S camera and a Samsung Galaxy S6 combined with Samsung GearVR goggles (Figure 1). Moreover, several free available VR apps especially designed for VR goggles were installed. Patients were free to use them next to the livestream. Patients received VisitU for 3-5 days during their hospitalization. Before the start of the experiment, the researcher (AB) briefly instructed the patients on the use of VisitU. Furthermore, written instructions were available on paper and online. In practice, patients utilized the Samsung Galaxy S6 and the Samsung VR goggles in the hospital, and parents used the laptop and 360° camera at home. When the camera was connected to the laptop, a virtual meeting could be created. Subsequently, an email invitation was sent to the smartphone, and by accepting the invitation, the live VR meeting would start automatically. The smartphone was then placed in the VR goggles, and patients could experience the virtual visit to their home. During the research period, VisitU updated their service and thereafter, the VR livestream opened directly through an app, and accepting the email invitation was no longer necessary.

### Data Collection

Data collection started with including patients who, in consultation with the pedagogical staff, were approached by the researcher. One patient at a time was included because only one VR device was available for research. Informed written consent was obtained from all subjects. Subsequently, 2 semistructured interviews were conducted—one before and one after the period in which the device could be used. The qualitative data were collected by audiorecording the interviews. In addition, patients or their parents were asked to keep a diary on the use of the VR intervention. With regard to the interview, a semistructured interview guide was developed concerning the background information, experiences with VisitU in different domains, and relevant factors found in the literature on VR and videoconferencing (Multimedia Appendix 1). The interview guide was discussed among the research team and evaluated after every couple of interviews. The interviews were conducted by the researcher (AB) and preferably performed face-to-face in the hospital ward. We interviewed patients, parents, or other relatives. The duration and content of the interviews were adjusted to the age, sickness, and concentration of each patient. Consequently, in total, 15-25 minutes of interview data for each patient were recorded. Furthermore, verbal member check was performed at the end of every second interview.

### Data Analysis

The interviews were transcribed verbatim using audio transcription software F4 [28]. In addition, ATLAS.ti software was used to facilitate the analysis of the interviews [29]. The inductive thematic analysis was done according to the 6-step procedure described by Braun and Clarke [30] and it proceeded as follows. We started with familiarizing ourselves with the data by transcribing and rereading the interviews. Then, the process of open coding was performed, followed by revising the codes, also known as axial coding. To continue the inductive analysis, the open codes were grouped into categories. The categories were discussed among the research team and were subsequently collected in themes. The interim analysis was conducted to refine interview questions and estimate data saturation [31]. Of note, the data collected by the diary were not separately analyzed. The information obtained from the diary (ie, time of use and profits and barriers of the VR device) was used during the interviews.

## Results

### Study Group

A total of 10 children were included, and 18 interviews were performed in this study. With 1 child, based on medical conditions, we chose to perform only one interview after the use of VisitU. Another patient was lost to follow-up; therefore, the second interview could not take place. Of 18 interviews, 3 were with patients exclusively, 13 with both the patient and the parent, and 2 with a parent or relative only. The method of interviewing was face-to-face 15 times and in 3 cases, by telephone because of early or weekend discharge. All patients aged 9-15 years old (mean age: 11 years and 8 months); 8 boys and 2 girls were included. The time of hospitalization was 5 days to >4 weeks (median 12 days), and the average hospital to home distance was 55 km (range 3-100 km). Of 10 patients, 3 were treated in isolation during hospitalization. The specialty of care varied (surgery, neurology, pulmonology, oncology, infectious disease, and cardiology), and the number of hospitalizations ranged from 1 to 3 over the last year.

### Overview

The interviews were transcribed with a total of 27,531 words from which 79 codes were made. After interviewing 8 children, no new codes came up from the interviews. After analysis, the codes were subdivided into 18 categories and collected into 6 themes as follows: “Being hospitalized,” “Expectations of VisitU,” “VisitU in use,” “VisitU, the profits,” “The impact of VisitU,” and “Barriers when using VisitU.” The first 2 themes represent the patients’ view and experiences before the use of VisitU, and the last 4 themes represent the experiences with VisitU after usage. Table 1 shows an overview of the themes and categories with corresponding quotes.

### Being Hospitalized

The impact hospitalization, ways of coping, and social connection were discussed. A majority of the patients (7/10) stated they did not want to be hospitalized. A variety of reasons were expressed such as physical discomfort, boredom, and uncertainty. Moreover, being obliged to lay in bed, to stay in the hospital, and to take drugs was bothersome. The wish for the presence of friends and family around them was expressed by half of the patients. The absence of their pets was additionally named by 2 patients. The children longed for participating in their usual activities, such as going to school, going on a vacation trip, or simply being at home. To deal with their hospitalization, different methods of distraction were used such as gaming or drawing. To connect with friends or other relatives, patients received visits in the hospital or were digitally in contact by texting or video calling. Contacts with the hospital staff and sources of entertainment, such as electronics, were mentioned as positive aspects of hospitalization.

### Expectations of VisitU

Along with discussing expectations of VisitU, patients were questioned about previous experiences with VR. Opinions differed as to whether the use of VR earlier has been a satisfying experience. Although a minority of the patients (4/10) had previously used a VR device, all had an idea of the purpose and utilization of VR goggles; their expectations varied. Some patients expected to look around at home or in a virtual world, whereas they were not really there. Other expectations were the 360° look around in, the 3D effect of the VR goggles or “just something new.”

### VisitU in Use

There was a range in time, location, person, and content concerning the actual use of the VR system. The usage of the VR goggles differed from once to multiple times a day. The duration ranged from 1 minute to multiple hours, and the median duration was 15 minutes each time. The laptop and camera were most often brought home, set up by one of the parents, and installed at one usual place. In the hospital, both patients and their friends and other relatives used the VR goggles. When using the VR livestream, patients talked with relatives at home, observed their daily activities, or gazed around in the house. In practice, 7 of 10 patients used the VR livestream, and 6 of them utilized other VR apps. One patient did not use the VisitU device at all; instead, 2D livestream was used. In the case of using other VR apps, patients played with free available VR apps, such as a rollercoaster app, or watched VR videos on YouTube.

### VisitU: The Benefits

The main benefit, according to almost all patients, was the ability to be somewhere else through the VR goggles. The view of the hospital surroundings was blocked through the head-mounted display, and patients said they were, therefore, “not being here but there.” As a result, patients were offered a way to escape the hospital. Not only being somewhere else was mentioned as a benefit but also, in particular, the opportunity to virtually be in their own houses (5/6). In addition, VisitU facilitated social connection with relatives at home. Children could easily talk to their parents, siblings, or friends and be part of their “normal lives.” The distraction VR created was another benefit that 3 of 8 patients and relatives reported. It was just something different than the hospital, and patients enjoyed playing with the VR device.

### The Impact of Using VisitU

When talking about their thoughts on VR livestream, all patients’ reactions were positive. There was a range in enthusiasm from “I thought it was quite nice” to “Very enjoyable and cool!” When asked, patients were unanimous in the view that they would like to use VisitU again and would recommend it to other patients. Patients said that VisitU made them feel happy; for example, one mother said, “As soon as he puts on the VR goggles, a big smile appears on his face.” Noteworthy is the comment of one patient that he felt “depressed” right after the usage because he did not want to quit. Some parents and patients said they also used alternative technical devices like WhatsApp or video calling to fulfill their needs for connection. For another patient, the VR games did not meet his expectations. Part of experiencing VR is the perception of being physically present in the virtual world. All patients said the virtual world felt real to them in some way. The presence of the virtual world was surprising for one patient; she described it as “First, I could see my parents and the next moment, when taking the VR goggles off, I was back in the hospital.” The sounds of the hospital and the impossibility to touch their relatives at home were mentioned to decrease the sense of reality.

**Table 1:** The qualitative analysis.

Theme and category		Quote (example)^a^
**Being hospitalized**		
	Impact of hospitalization	Interviewer: What’s it like to be in the hospital? 8aChildB09: I don’t like it. Actually, I don’t want to be here.
	Social connection during hospitalization	9aChildB15: I would rather be at home, because there I can be among all my friends and everything. I can’t do that so much here.
	Coping with hospitalization	4aChildB11: Yes, you can play and sometimes people come and play with you.
**VisitU in use**		
	Ways of use VisitU	5bChildB09: The whole evening really, I used it quite a lot.
	Using other VR^b^ apps	1bChildB14:...the extra apps on it were also quite fun to use…at one point I could see the T. rex eat from, I didn’t see what really, but I could literally stand below them while they were eating.
**VisitU: the benefits**		
	Being somewhere else	2bChildB14: Just to be in a different place...So, you’re away from the hospital.
	Being home	9bChildB15:...so you also get a sense of knowing what it looks like at home and what has changed and everything.
	Being connected	1bChildB14: I saw our pets again for a little while; it was nice to see them again for a moment. I do see them every now and again with WhatsApp, but then [with VR glasses] I saw them better...at one point she [dog] also looked and pushed her little nose up against the camera so I was able to see her again.
	Being distracted	10bChildG14: It does help to take your mind off being in the hospital a little bit.
**Barriers when using VisitU**		
	Technical reliability	5bChildB09: So, I mean, the patient can, for example, only see the parents. If the parents could also see the child, it would be a bit better, but that will be difficult to create, I think.
	Complexity	5bMotherB09: I installed it at home [laughs]...I thought it was still quite complicated, as you obviously have to create a moment every time.
	Physical effects	3bChildB10: But after having played with these goggles a lot, my head hurts. I don’t feel dizzy; my head just hurts.
**Impact of using VisitU**		
	Reality of VR	10bChildG14: Yes, home is different, because it’s just a bit different really…so grandma has a glass door and often you can see yourself in it, but when I turned around in it, when I turned toward it [with VR goggles], I didn’t see myself [in the glass].
	Feelings on VisitU	9bChildB15**:** Then it´s less hard to be here in the hospital.
	Thoughts on VisitU	7bSister13B09: Yeah I do think I would recommend it...

^a^Codes used for quotation consist of the number of a patient, a letter “a” for first and “b” for second interview, the role of the quoted person, gender, and age; for example, 3bChildB10 is the 3rd child, second interview (b), patient himself, boy (B) and 10 years old.

^b^VR: virtual reality.

### Barriers When Using VisitU

Although most patients were enthusiastic about the idea of VisitU, some of them experienced technical, practical, or physical limitations. The one-way connection of the VR livestream was seen as a disadvantage by half of the parents or patients. At home, they could only hear the patient in contrast to the patient who could also see the other people. On the quality of the resolution of the VR goggles, opinions differed, and an upgrade of the display resolution was suggested. The high temperature of the telephone when using the VR goggles was also noticed by one patient. Besides these disadvantages, 2 parents had to deal with temporary technical problems as a result of an outdated version of VisitU and a problem with the camera software. The unfamiliarity with VR and the system was brought up as a barrier, and 3 of 6 parents thought that the installation was complex and time consuming. Patients, in contrast to their parents, thought the smartphone and VR goggles were easy to use. More than half of the patients (5/8) experienced physical side effects when using VR for a while. Symptoms were experienced after a range of only 1 minute to half an hour of use. Patients mentioned side effects such as a headache, nausea, and dizziness; these symptoms disappeared when the VR livestream was interrupted. Subsequently, most patients continued the use of VisitU despite experiencing symptoms.

### Challenges for the Hospital Staff

Concerning the technical reliability during the research period, 2 software problems were noticed. The first problem was the need of a software update, and the second problem relating to the camera and teleporting system occurred. On the other hand, the organization was a challenge for the researchers. Scheduling a meeting with each patient to hand over VisitU was difficult as a consequence of unplanned care in a hospital ward.

## Discussion

### Principal Findings

**Figure 2:**
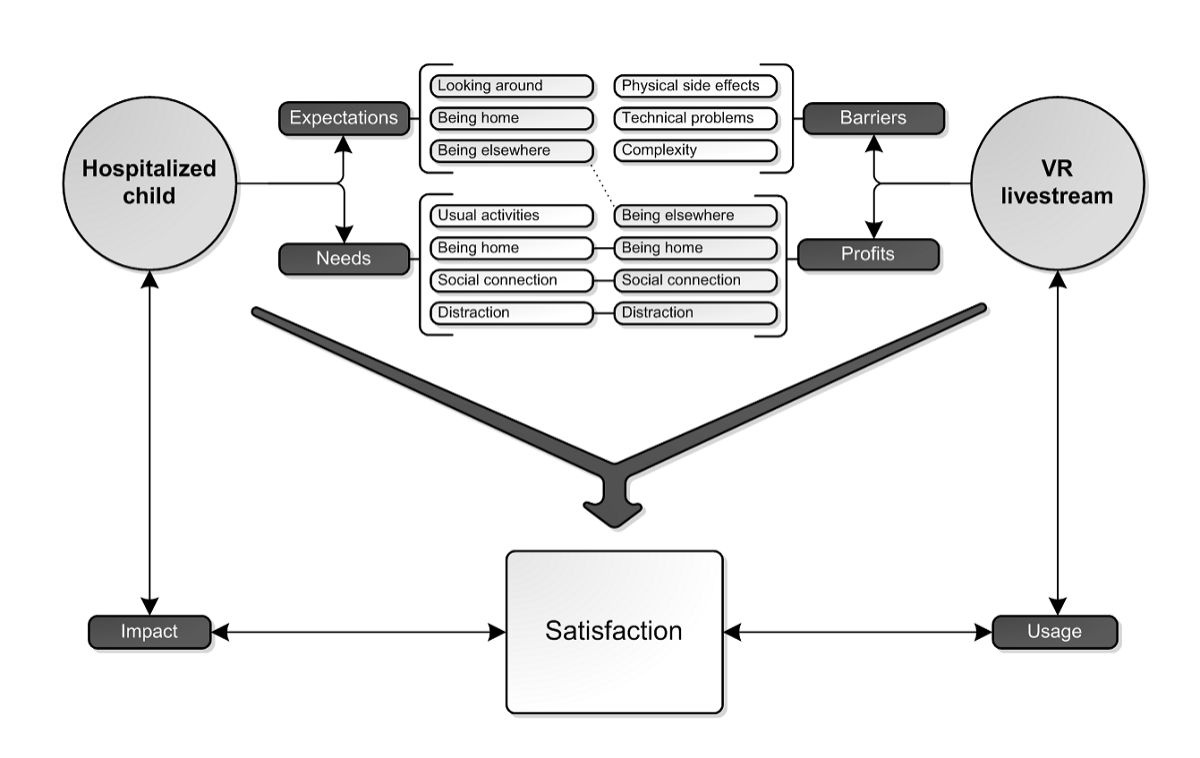
Model of experiences with VR livestream.

This study shows that pediatric inpatients were positive about the idea, effect, and possibilities of VR live streaming. Barriers were experienced on the technical abilities, physical side effects, and complexity of usage. In addition, the research shows that hospitalized children long for participation in their usual activities, social connection, and distraction. Being somewhere else, being at home, and facilitating social connection were the main benefits of VisitU. Therefore, VisitU matches the needs of these hospitalized patients. Although the way of use by patients varied, all patients would recommend VisitU and would use it again. Figure 2 presents a hypothesized model of the experiences with VR livestream based on these results. We assume that both the expectations and needs, as well as the experienced barriers and benefits, affect the degree of satisfaction of the product and consequently, affect the usage and impact of VR livestream.

### Comparison With Prior Work

As far as we know, this is the first study that focuses on VR livestream. Therefore, the results are unique and cannot be compared directly to previous literature. Therefore, in this section, the findings will be compared with the literature on either videoconferencing or VR separately. The observations in this study support the hypothesis that combining those 2 techniques in one device is valuable for hospitalized patients. Prior studies about videoconferencing and VR have noted similar effects as our study regarding the feelings and thoughts of users [7-9,12]. Patients felt more positive and felt more “normal” when they used videoconferencing [7,8]. In addition, VR improved the emotional state and positive emotions during hospitalization [12]. Therefore, it is likely that the combination of VR and videoconferencing, as the results of this study suggest, also causes positive feelings and improves the experience of hospitalization.

Regarding the main benefits of VisitU, the finding that live streaming facilitates social connection agrees with the findings of Nicholas et al’s findings evaluating videophone communication; their study showed that patients felt less lonely, frightened, and stressed when talking with family and friends on the videophone [8]. In addition, in a feasibility study of VR in the hospital, being somewhere else was also found to be a benefit. Here confirming our findings, a patient described VR provides an “escape” from the confines and boredom of the hospital room [23].

Little is known about using VR interventions unrestricted in a hospital setting, like VisitU was used. In nearly all studies about videoconferencing and VR, the actual use was either one-time or regulated. Only a few studies reported the efficacy of repeatedly using VR. Our results are in line with their suggestions that VR stays effective after repeatedly using it within a couple of days [20,32].

The presence of technical and practical barriers mentioned by patients in this study seems to be consistent with previous research on electronic health (eHealth) and VR [33,34]. Technical reliability is one of the main challenges when implementing an eHealth program [33]. Along with the technical problems, Eysenbach hypothesized that usability, ease of enrollment, workload, and time required are other factors obviously affecting the usage of eHealth [35]. These themes are comparable to the factors mentioned in this study. The side effects match those observed in former VR studies. More than half of the patients reported side effects. In a recent systematic review of VR for hospitalized patients, only 17% of patients experienced side effects. In contrast, another study reported symptoms in 80% of VR users [12,22,36]. No clear cause exists for this variety in the frequency of side effects. Cyber sickness is known to be a result of accumulating factors, including the duration of exposure to VR. Therefore, unrestricted use may be the reason a majority of patients experienced side effects [37]. In addition, as a consequence of being hospitalized for a longer period, this specific population could be more sensitive to side effects. Furthermore, technical adjustments to VR devices can reduce symptoms of cyber sickness [36]. Hopefully, these techniques will decrease the number of side effects in the near future [36].

### Strengths and Limitations

The setting and design are the key strengths of this study. An explorative qualitative approach and inductive analysis were chosen as design to focus on the patients’ perspectives and understand why and if VisitU would be a useful innovation. In addition, the setting was a tertiary hospital with patients hospitalized for mostly a longer period and a relatively far distance from home. Therefore, VisitU was relevant for this specific population.

Despite the strengths, this study also has several limitations. The major limitation of this study is the risk for the researcher bias because the process of coding was only done by one researcher (AB). To overcome this limitation, the codes and analysis were discussed on a regular basis among the research team (LJ and CN) [38]. To ensure external reliability, the raw data, transcribed interviews, and codes are well documented and transparent [39]. The method of triangulation using a diary was barely used by patients. To overcome this limitation, the information was asked in the interviews. Finally, a purposive sample was chosen to improve the external validity; unfortunately, the boy-to-girl ratio is unequal, and it is not known if this affects the results [39].

### Recommendations

Although this study is based on a small sample of participants, the findings suggest VisitU brings together the needs of patients and the possibilities of VR and videoconferencing. VisitU seems to be feasible in hospital wards, and we recommend the implementation together with further development and evaluation. Further work is required to improve the reliability of the VisitU technology and the usability of the system. Along with developing VisitU, more research is needed on VR live streaming in other pediatric populations and on different implications of VisitU, such as intensive care units or isolation rooms. Furthermore, further research is required to reduce the side effects of VR. Children do not like being hospitalized; therefore, other innovative ways to improve the experiences with hospitalization are also a field of further research.

### Conclusions

This study shows the positive experiences of children and adolescents with VR live streaming. The results suggest that VR can improve the experiences with hospitalization in pediatric patients. VisitU offers patients a way out of the hospital. It meets the needs of patients for being at home, socially connected, and distracted during hospitalization. Nevertheless, technical and practical barriers must be overcome, and further studies must be performed to understand the side effects of VR.

## Appendix

Multimedia Appendix 1 Interview guide.

## Abbreviations

eHealth: electronic health
VR: virtual reality
