# Supplementary material for: Home to Hospital Live Streaming With Virtual Reality Goggles: A Qualitative Study Exploring the Experiences of Hospitalized Children
Source: JMIR Pediatr Parent. 2018 Dec 13;1(2):e10. doi: 10.2196/pediatrics.9576 (PMC6716480; doi:10.2196/pediatrics.9576)
Supplement: Multimedia Appendix 1 [file pediatrics_v1i2e10_app1.pdf]

Appendix I

Interview guide

Background

Topic

- Sub topic
    - Sample question

The order and content of the questions was adjusted to the age, sickness and concentration of each patient.

Interview A: before start of the experiment

- ❖ Introduction
    - Explanation research and interview
- ❖ Experience with hospitalization
    - Hospitalization frequency
        - Have you ever been hospitalized before? Can you tell me about that?
    - Reason for hospitalization
        - Do you know the reason for your hospitalization?
    - Experiences with hospitalization
        - What do you think about your hospitalization? What is it like to be in the hospital?
    - Interventions during hospitalization
        - What do you like in the hospital? What kind of things do you want to do in the hospital?
- ❖ Home
    - Not being home
        - What is it like not being at home?
    - Connection
        - Do you contact others while you're hospitalized? In what way?
- ❖ Virtual Reality
    - Experience
        - Have you used a VR device before? What was it like?
    - Expectations
        - What is your idea about the use of VisitU?
- ❖ Verbal member check
- ❖ Conclusion

Interview B: after the period in which the device could be used

- ❖ Introduction
    - Explanation research and interview
- ❖ Experience with hospitalization
    - Any question missed during interview A.
- ❖ Virtual Reality experiences
    - Usage
        - How often have you used the VR device? In what way have you been using VR? When did you use VR?
    - Practical
        - What was the usability of the VR goggles?
    - Physical
        - What happened with you physically when you used the VR goggles?
    - Presence
        - How real was the VR environment? In what way did you consider yourself being there?
    - Cognitive
        - What was using the VR goggles like? What thoughts came up in your mind? What would you tell your friends about these goggles?
    - Emotional

    - What kind of feelings did you experience using VR?

  - Social
    - Whom did you contact through VR goggles? What were your thoughts about that?
  - Expectations
    - Did VR live stream meet your expectations?
- Parents view
  - Cognitive
    - What were your thoughts about the of VR use for your child?
  - Emotion
    - In what way affected VR your child?
  - Behavior
    - How did your child react using VR?
  - Practical
    - What were your experiences with using and installing VR live stream?
- Verbal member check
- Conclusion
